# The National COVID Cancer Antibody Survey: a hyper-accelerated study proof of principle for cancer research

**DOI:** 10.1038/s41416-023-02251-9

**Published:** 2023-04-20

**Authors:** Matthew Fittall, Justin Liu, James Platt, Maria Ionescu, Remarez Sheehan, Sukhmunni Johal, Rosie Mew, James Clark, Izzy Watts, Arvind Tripathy, Martin Little, Grisma Patel, Hari Panneerselvam, Nathan Appanna, Emma Burke, Hayley McKenzie, Michael Tilby, Sam Khan, Lennard Y. W. Lee

**Affiliations:** 1 Cancer Institute, University College London, London, WC1E 6DD UK; 2 Leeds Institute of Medical Research, University of Leeds, Leeds, LS9 7TF UK; 3 UK Health Security Agency, Fleetbank House, London, EC4Y 8AE UK; 4 Oxford University Hospitals, Oxford, OX3 9DU UK; 5 Royal Devon University Healthcare NHS Foundation Trust, Exeter, EX2 5DW UK; 6 University College Hospitals NHS Foundation Trust, London, NW1 2PG UK; 7 University Hospitals of North Midlands NHS Trust, Stoke-on-Trent, ST4 6QG UK; 8 Maidstone and Tunbridge Wells NHS Trust, Kent, TN2 4QJ UK; 9 Wye Valley NHS Trust, Kent, TN2 4QJ UK; 10 University Hospital Southampton NHS Foundation Trust, Hampshire, SO16 6YD UK; 11 University Hospital Coventry, UHCW NHS Trust, Coventry, CV2 2DX UK; 12 University of Leicester, Leicester, LE1 7RH UK

**Keywords:** Adaptive clinical trial, Epidemiology, Prognostic markers

## Abstract

The COVID-19 pandemic has led to a range of novel and adaptive research designs. In this perspective, we use our experience coordinating the National COVID Cancer Antibody Survey to demonstrate how a balance between speed and integrity can be achieved within a hyper-accelerated study design. Using the COVID-19 pandemic as an example, we show this approach is necessary in the face of uncertain and evolving situations wherein reliable information is needed in a timely fashion to guide policy. We identify streamlined participant involvement, healthcare systems integration, data architecture and real-world real-time analytics as key areas that differentiate this design from traditional cancer trials, and enable rapid results. Caution needs to be taken to avoid the exclusion of patient subgroups without digital access or literacy. We summarise the merits and defining features of hyper-accelerated cancer studies.

Cancer clinical trials have developed into a diverse and sophisticated array of designs suited to differing purposes. These trials aim to give robust insights into cancer biology, pathology, investigation and management. The heritage of these trials, developed prior to the utilisation of electronic health records and population-scale datasets, persists in modern clinical practice.

The speed of the COVID-19 pandemic and its potential threat to society inspired a revolution in clinical trial design, achieved through collaboration and innovation between clinical specialties, research organisations, academic institutions and governments. A greater emphasis was placed on expedited and pragmatic studies, so termed hyper-accelerated trials. These novel approaches encompassed clinical testing, risk stratification and the development of therapeutics: the RECOVERY programme rapidly established a coordinated national clinical trial assessing repurposed therapeutics in hospitalised patients [1], the FALCON Moonshot C-19 studies provided the validation for lateral flow tests in the UK [2] and the national coordination of testing, hospitalisation data and viral sequencing afforded rapid insights into viral evolution and clinical risk [3, 4]. The development of vaccines has been well described and rightly lauded [5]. The MHRA has accepted the findings from these new study designs, which has led to new diagnostics, therapeutics and vaccines being approved in record time.

The COVID-19 pandemic has had a significant impact on cancer care, with delayed diagnoses and treatments, and disruption to research. Cancer patients have also been more directly impacted by COVID-19 than the general population [6, 7]. This risk was identified through the efforts of the UK Coronavirus Cancer Programme (UKCCP), one of the longest running cancer and COVID-19 research programmes globally [8].

A major clinical concern at the end of 2021, and the rationale for launching the National COVID Cancer Antibody Survey, was whether cancer patients received as much protection from COVID-19 vaccination as the general population. The survey therefore aimed to appraise the utility of assessing immune responses in cancer patients post-vaccination through antibody testing. In 2021, pivotal immunology phenotyping studies were published from UK research consortiums such as CAPTURE [9] and SOAP [10]. Similar validation studies were also published in each cancer subtype, though with limitations. These studies used varying assays and meant there was no way to compare antibody responses across the cancer population. Most crucially, there was no known link between antibody response and COVID-19 protection. The National COVID Cancer Antibody Survey was an ambitious attempt to address these issues in a timely way to inform the ongoing vaccination programme.

The National COVID Cancer Antibody Survey was launched in September 2021, offering COVID-19 antibody testing to any cancer patient in their own home anywhere in England. Results were returned to both the patient and their primary care provider within one week. Testing data were immediately available for integration with national COVID-19 testing and hospitalisation datasets and cancer diagnostic and treatment registries. By March 2022, over 3,500 individuals had been tested with a draft report issued in April 2022.

There were four areas in which the survey, a hyper-accelerated study, differs from more traditional cancer clinical trial design (Fig. 1).

**Fig. 1:**
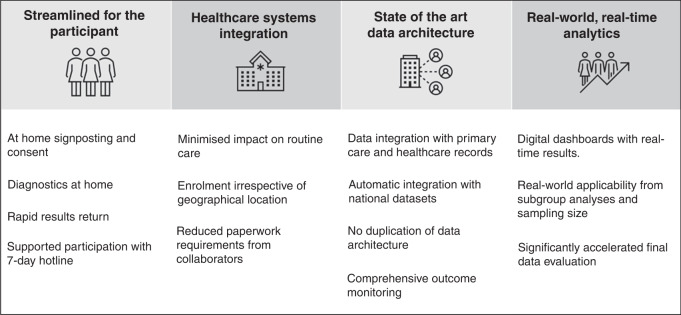
Acceleration points by pandemics studies. This diagram shows how these points were used by the National COVID Cancer antibody survey.

## Streamlined for the participant

The survey was designed in conjunction with patient charities and groups, directly responding to the needs of the community as well as national priority. Twenty-four charities worked with the study delivery team and they provided signposting of the study to sites, maximising recruitment. Once individuals were aware of the study, they were able to access all information and provide consent at home. The intervention for this study was then performed from the comfort of the patient’s home using the Home Antibody Testing Service from the Department of Health. This was important to minimise the risk of exposure to COVID-19 within the hospital setting or during travel. Capillary blood samples were processed at centralised pandemic response laboratories. Results were made accessible in near-real time, with a 7-day hotline and support from clinicians to address patient queries. As a result of study participation, patients had accelerated access to COVID-19 antibody testing and were able to contribute to crucial national research.

## Healthcare systems integration

**Fig. 2:**
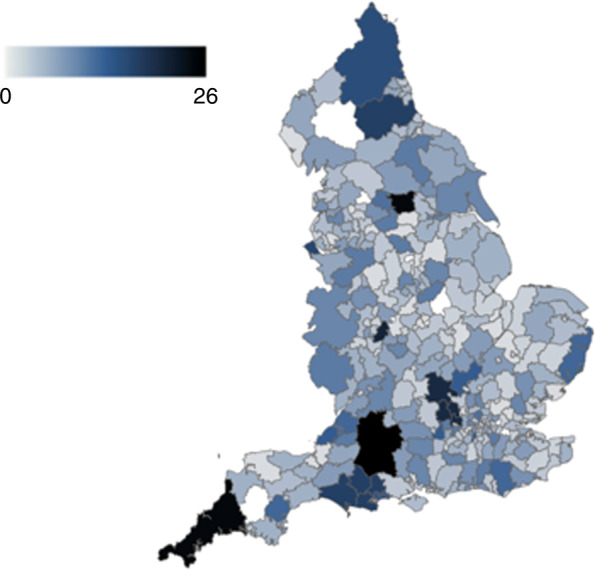
Geographical map showing lower layer super output area (LSOA) where patients accessed COVID-19 antibody testing and were included in the National COVID Cancer Antibody Survey. Density is represented by darker shades as shown in the key.

The corollary of this distributed patient-led approach is that it was independent of healthcare systems, not putting any additional strain on their resources. Recruitment was not limited to local or regional institutions and had minimal negative impact on routine clinical care. The National COVID Cancer Antibody Survey map illustrated extensive geographical reach across the UK, including rural and deprived areas. It reached well beyond regions normally serviced by academically active NHS sites or regions with large trials units (Fig. 2).

## State-of-the-art data architecture

Antibody testing results were simultaneously made available to primary care physicians and accessible within patients’ own care records. Study data were linked to existing national secondary care datasets, limiting duplication of effort and facilitating comprehensive outcome monitoring for both subsequent COVID-19 infection and hospitalisation.

## Real-world, real-time analytics

Underpinning trial operations, a separate data and analytics taskforce established a pipeline for real-time assessment of results. Data monitoring committees were able to assess study outcomes through state-of-the-art “dashboards” that were automatically updated based on primary outcomes, secondary outcomes and subgroups. Therefore, once accrural was complete, undertaking the final analyses was significantly accelerated.

## Discussion

The UK COVID Cancer Antibody Survey recruited 3555 patients in six months, with uptake across all regions of England, and prompted extremely positive feedback from participating clinicians, charities and patients. The main limitation of our study design was the potential for digital exclusion, as patients were approached and consented online. Consent at home requires access and familiarity with personal electronic devices. On reflection, while attempts were made to develop user-friendly websites, a lack of analogue telephone capabilities likely limited participation. Hence, such facilities should be made available for future hyper-accelerated studies. While our study was observational in nature we believe there are lessons to be learnt across other types of cancer studies.

Evaluating new methods of population cancer screening is the most amenable to a hyper-accelerated design and the NHS-Galleri trial of the use of a multi cancer early detection test already exemplifies this [11]. The trial focused on patient accessibility, and the use of existing data infrastructure, such as cancer registries and primary and secondary care datasets, to expedite recruitment and data handling with greater cost efficiency [11]. Rectruitment and screening was independent of the healthcare system, reducing burden, with patients patients only integrating into routine diagnostic pathways with a positive result. This study achieved its recruitment target of 140,000 patients within 10 months in 2021–2022, which is a testament to the study’s successful design and acceptability to the target population.

Studies to improve the diagnosis and stratification of cancer may also benefit from improved patient recruitment, data integration and analysis, particularly where subtypes are rare. The angiosarcoma project is one such example, which aimed to improve the diagnosis and understanding of a disease which represents only 0.01% of cancer diagnoses annually [12]. Charity and direct patient involvement patients supported self-enrolment and online consenting resulting in the recruitment of 338 patients in 18 months. This is the largest cohort of patients with angiosarcoma assembled to date. The cohort represented a wide geographical area of North America, including populations that do not typically contribute to academic studies.

Cancer intervention studies clearly provide the most challenge for hyper-acceleration because of their need to carefully control both intervention delivery and precise outcome measurement. The greater utility of molecular features to define treatment suitability will, however, necessitate improved data integration, particularly where a target for therapy is rare but not cancer type specific. This is relevant in the field of mRNA cancer vaccines, where the success of COVID-19 vaccines and recent efficacy data published by Moderna has refocused UK policy leading to an ambitious target to vaccinate 10,000 cancer patients by 2030 [13]. The feasibility of individualised cancer vaccine generation relies on the collection of genetic sequencing information which is not a routine part of diagnostic pathways. National integration of distributed healthcare systems and data architecture could be vital for rapid collation, analysis and turnover of results.

The structure and design of clinical trials has evolved significantly since the start of the COVID-19 pandemic. The scale of threat and pace of viral evolution required researchers and institutions to focus on efficiency and speed of reporting, whilst maintaining the highest quality and statutory compliance. This rebalancing of requirements has facilitated a re-thinking of every part of clinical studies including recruitment, consent, intervention, patient support and analysis, and has resulted in this new paradigm of hyper-accelerated studies. Applying these changes to cancer research more broadly will be challenging but national studies like ours and the Galleri screening study demonstrate the protential benefits.

## Data Availability

The present manuscript is a qualitative exercise and therefore does not reference raw datasets.
